# Differential Stability of Flamingo Protein Complexes Underlies the Establishment of Planar Polarity

**DOI:** 10.1016/j.cub.2008.08.063

**Published:** 2008-10-28

**Authors:** Helen Strutt, David Strutt

**Affiliations:** 1 MRC Centre for Developmental and Biomedical Genetics and, Department of Biomedical Science, University of Sheffield, Western Bank, S10 2TN Sheffield, United Kingdom

**Keywords:** DEVBIO, CELLBIO

## Abstract

**Background:**

The planar polarization of developing tissues is controlled by a conserved set of core planar polarity proteins. In the *Drosophila* pupal wing, these proteins adopt distinct proximal and distal localizations in apicolateral junctions that act as subcellular polarity cues to control morphological events. The core polarity protein Flamingo (Fmi) localizes to both proximal and distal cell boundaries and is known to have asymmetric activity, but the molecular basis of this asymmetric activity is unknown.

**Results:**

We examine the role of Fmi in controlling asymmetric localization of polarity proteins in pupal wing cells. We find that Fmi interacts preferentially with distal-complex components, rather than with proximal components, and present evidence that there are different domain requirements for Fmi to associate with distal and proximal components. We further show that distally and proximally localized proteins cooperate to allow stable accumulation of Fmi at apicolateral junctions and present evidence that the rates of endocytic trafficking of Fmi are increased when Fmi is not in a stable asymmetric complex. Finally, we provide evidence that Fmi is trafficked from junctions via both Dishevelled-dependent and Dishevelled-independent mechanisms.

**Conclusions:**

We present a model in which the primary function of Fmi is to participate in the formation of inherently stable asymmetric junctional complexes: Removal from junctions of Fmi that is not in stable complexes, combined with directional trafficking of Frizzled and Fmi to the distal cell edge, drives the establishment of cellular asymmetry.

## Introduction

**Figure 1:**
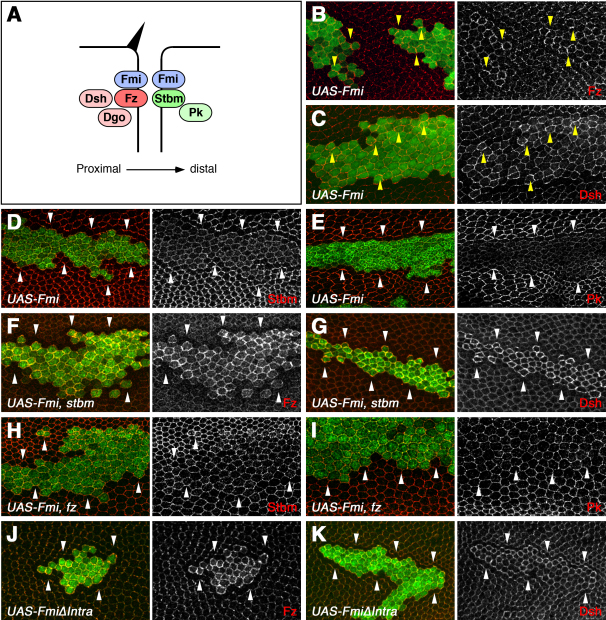
Overexpression of Fmi (A) Cartoon illustrating the asymmetric localization of planar polarity proteins during pupal wing development. (B–I) Pupal wing clones overexpressing Fmi, marked by lacZ (B and C) or Fmi (D–I) staining in green. (B–E) *UAS-Fmi* clones in a wild-type background stained for Fz (B), Dsh (C), Stbm (D), or Pk (E) in red. Yellow arrowheads (B and C) point to cell-cell boundaries internal to the clone with an accumulation of Fz or Dsh staining. (F and G) *UAS-Fmi* clones in a *stbm^6^* mutant background, stained for Fz (F) or Dsh (G) in red. Note that the increase in junctional staining is more intense. (H and I) *UAS-Fmi* clones in a *fz^21^* mutant background, stained for Stbm (H) or Pk (I) in red. Note that the cells above the clone in (H) are smaller as they are in the wing vein. (J and K) Pupal wing clones overexpressing FmiΔIntra, marked by Fmi staining in green, and stained for Fz (J) or Dsh (K) in red. Pupal wings are shown with distal to the right, in this and all subsequent figures. White arrowheads point to clone boundaries.

Understanding the mechanisms by which cellular asymmetry is generated within the plane of an epithelium is a fundamental problem in biology. One system in which this has been well studied is the planar polarization of the *Drosophila* wing. In this tissue, each cell produces a single distally pointing actin-rich trichome from its apical surface. The trichomes begin to emerge from the distal side of wing cells during pupal development, and key to the decision about the site of hair formation is the prior assembly of an asymmetric protein complex in the apicolateral plasma membrane (Figure 1A). Specifically, the seven-pass transmembrane protein Frizzled (Fz) localizes to the distal end of each cell, at the site of trichome formation, where it associates with the cytoplasmic proteins Dishevelled (Dsh) and Diego (Dgo). Conversely, the four-pass transmembrane protein Strabismus (Stbm, also known as Van Gogh) localizes to the opposite side of the cell, together with the cytoplasmic protein Prickle (Pk). Finally, another seven-pass transmembrane protein Flamingo (Fmi, also known as Starry night) is thought to localize both proximally and distally. Loss of any of these core planar polarity proteins causes a loss of the asymmetric distribution of the other members of the complex, which leads to defects in trichome placement (reviewed in [1, 2]).

The mechanisms by which this asymmetric distribution is achieved are poorly understood. Early in pupal life, asymmetry is not apparent, but it subsequently becomes visible by 24–28 hr after puparium formation (APF), shortly before the trichome emerges [3]. It has been suggested that an initial asymmetry in the activity or distribution of one or more of the planar polarity genes is subsequently amplified by interactions between the polarity genes themselves [4, 5]. The nature of this initial asymmetry has not been identified, but it is thought to be the result of long-range patterning cues within the proximal-distal wing axis. In particular, the atypical cadherin molecules Dachsous and Fat, and the Golgi protein Four-jointed, have been widely thought to act upstream of Fz to provide global patterning information (reviewed in [6, 7]), although there is increasing evidence against this (e.g., [8–10]).

Interactions between polarity proteins may occur both intracellularly and intercellularly across the apicolateral junctions. In particular, clones of cells lacking Fz or Stbm activity exhibit nonautonomous effects on the polarity of neighboring cells, consistent with these factors mediating intercellular communication [11–13]. In addition, although clones of cells lacking Fmi activity do not show nonautonomy [3, 14], recent data suggest that Fmi is required on both sides of the cell boundary for Fz/Stbm-dependent cell-cell communication [15, 16]. Interestingly, the N-terminal extracellular domain of Fmi contains multiple cadherin repeats, which have been demonstrated to interact homophilically in cell culture, and thus could mediate communication between proximal and distal polarity proteins within adjacent cells [3].

How the polarity proteins might interact to achieve asymmetric localization is poorly understood. Analysis of polarity gene mutants has established that Fmi is a key determinant in the localization of the other polarity proteins to the apicolateral junctional region. In the absence of Fmi activity, Fz fails to be recruited to apical junctions, and Stbm levels at junctions are drastically reduced. Loss of Fz activity leads to a loss of both Dsh and Dgo from junctions. Finally, loss of Dsh, Dgo, or Pk activity does not affect Fz, Stbm, or Fmi apical recruitment but does block their asymmetric distribution [3, 5, 17–22]. Various in vitro interactions between members of the proximal and distal complexes have been identified, leading to the suggestion that asymmetry is promoted via competitive inhibition of access to binding partners [5, 23]. Indeed, mathematical modeling of such interactions has successfully simulated the observed in vivo asymmetry [4]. A second class of mathematical model has also suggested that proximal and distal complexes compete for access to Fmi [24]. In addition, Fmi homophilic interactions have been suggested to be stabilized by proximal and distal-complex components [1].

Interestingly, although Fmi appears to localize both proximally and distally, experimental evidence indicates that it can have asymmetric activity. Overexpression of Fmi causes a similar phenotype to a *fz* loss-of-function clone, such that trichomes point toward the clone [3, 18]. The *fz* clone phenotype can be explained the preferential localization of Stbm inside the clone to clone boundaries, where it can interact with Fz in cells outside the clone. Thus, on the distal side of the clone, the normal asymmetry of the polarity complex (and hair polarity) is reversed. The similar phenotype seen when Fmi is overexpressed is consistent with the preferential interaction of increased Fmi inside the clone with Fz-containing complexes in cells outside the clone. Hence, Fmi apparently does not exhibit identical affinities for distal and proximal complex components. Nevertheless, no molecular analysis of this asymmetric activity has been carried out to date.

There is also evidence that polarized transport of core proteins contributes to the generation of asymmetry. By live imaging of pupal wings, Fmi and Fz were shown to be present in intracellular vesicles that bud from junctions and are preferentially trafficked along microtubules toward distal cell edges, where they are reinserted into the plasma membrane [25]. Interestingly, Dsh has been implicated in regulating Fz internalization in vertebrate Wnt signaling [26, 27]. However, in planar polarity, Dsh (and also Pk and Dgo) has largely been thought of as a stabilizing factor, because its overexpression appears to cause polarity proteins to cluster at junctions [5, 18, 20]. Nevertheless, generation of asymmetry must require turnover and redistribution of the polarity proteins at junctions; thus, it is unlikely that Dsh, Pk, and Dgo merely “lock” the transmembrane proteins in place.

In this paper, we have investigated the molecular mechanisms controlling the asymmetric localization of the polarity proteins in pupal wing cells. We concentrate on the generation of molecular asymmetry, in which we consider how a protein complex forms with Fz:Dsh on one side of the cell-cell boundary and Stbm:Pk on the other, focusing particularly on the role of Fmi in this process. We find that Fmi binds preferentially to the Fz:Dsh complex and identify the C terminus of Fmi as important for promoting its association with Stbm:Pk. Moreover, we find that Fmi requires intercellular stabilization by proximal and distal-complex components at adherens junctions and that the stability of Fmi at junctions affects its rate of entry into endocytic trafficking pathways. Finally, our data suggest that trafficking of Fmi occurs via both Dsh-dependent and Dsh-independent mechanisms.

## Results

### Preferential Recruitment of Frizzled:Dishevelled to Junctions by Overexpressed Flamingo

In order to investigate the role of Fmi in the asymmetric localization of the polarity proteins, we overexpressed Fmi in clones in pupal wings and examined the subcellular localization of the other components. Interestingly, members of the proximal and distal complexes behave differently within such clones. Fz and Dsh are moderately stabilized not only at the clone boundary, but also at junctions between cells internal to the clone (yellow arrowheads in Figures 1B and 1C). In contrast, the levels of Stbm and Pk at junctions are reduced (Figures 1D and 1E), and a slight increase in cytoplasmic staining is observed. These effects are not due to alterations in transcription, because they are also observed with Fz or Stbm transgenes under control of heterologous promoters (Figures S1A and S1B, available online). Thus, when Fmi is overexpressed, it preferentially recruits Fz:Dsh to apical junctions rather than Stbm:Pk.

To characterize this effect more fully, we examined the effect of overexpressing Fmi in the absence of either proximal or distal-complex components. In the absence of *stbm* or *pk*, overexpressed Fmi further increases the levels of Fz:Dsh at junctions (compare Figures 1F and 1G to Figures 1B and 1C; and data not shown). These results are consistent with the possibility that both Fz and Stbm have an affinity for Fmi, but the interaction with Fz is preferred when Fmi is in excess. In the absence of Stbm, this preference is enhanced, and more junctional Fmi associates with Fz.

When Fmi is overexpressed in the absence of *fz* or *dsh* activity, then Stbm and Pk levels are no longer reduced at junctions (compare Figures 1H and 1I to Figures 1D and 1E; and data not shown). Importantly however, there is still no obvious stabilization of either protein above the normal level at junctions. The lack of increased recruitment of Stbm:Pk to junctions by overexpressed Fmi even in the absence of Fz:Dsh is unexpected. Fmi stabilizes Stbm at junctions in the wild-type wing, because Stbm levels are reduced in *fmi* mutant clones [18]. Furthermore, total cellular Stbm levels are unlikely to be limiting, because junctional levels of Stbm can be dramatically increased by overexpression of Pk or Dsh [18]. Therefore, it would appear that overexpressed Fmi is not competent to stabilize Stbm:Pk at junctions.

### The C-Terminal Domain of Flamingo Promotes Its Association with the Proximal Polarity Proteins

The differing ability of overexpressed Fmi to modulate Fz:Dsh and Stbm:Pk levels at junctions could be explained by a number of mechanisms. One likely hypothesis is that Fmi may require a cofactor for a robust interaction with Stbm, and that this cofactor is limiting when Fmi is overexpressed. Alternatively, Fmi may require posttranslational modification or a conformational change to interact with Stbm, and a factor needed for this modification is limiting. The cytoplasmic C-terminal tail of Fmi is a likely region to mediate an interaction with Fz:Dsh or Stbm:Pk; therefore, we generated a truncated form of Fmi, in which this region is either absent or replaced with GFP.

When overexpressed in pupal wing cells, FmiΔIntra is much more efficient at recruiting Fz and Dsh to junctions than full-length Fmi (compare Figures 1B and 1C to Figures 1J and 1K), an effect similar to that caused by removal of *stbm* or *pk* (Figures 1F and 1G). Stbm is still reduced at junctions—although less than when full-length Fmi is overexpressed (Figure S1C). This suggests that the C-terminal intracellular domain of Fmi is dispensible for the interaction of Fmi with Fz:Dsh and, importantly, that Fz:Dsh no longer have to compete with Stbm:Pk for access to Fmi.

Interestingly, two isoforms of Fmi have been identified, one of which contains a PDZ binding motif (PBM) at its C terminus. It is possible that loss of the PBM alone could account for the failure of overexpressed Fmi or FmiΔIntra to associate with Stbm:Pk. However, this is unlikely, because Fmi that lacks the PBM can rescue the planar polarity phenotype of *fmi* mutants [16].

**Figure 2:**
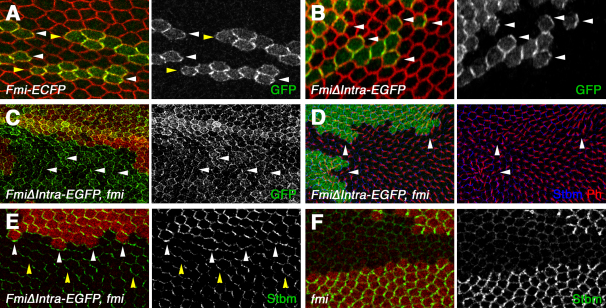
FmiΔIntra-EGFP Preferentially Interacts with Fz:Dsh (A and B) Clone of cells expressing Fmi-ECFP (A) or FmiΔIntra-EGFP (B) under control of the *Actin* promoter in the pupal wing, stained for GFP (green) and the junctional protein Armadillo (red). Fmi localization on distal cell boundaries is marked by white arrowheads and on proximal cell boundaries by yellow arrowheads. Note the predominantly distal localization of FmiΔIntra-EGFP. (C–E) Pupal wing clones of *fmi^E59^*, marked by loss of lacZ staining (red in [C] and [E], or green in [D]), in wings expressing FmiΔIntra-EGFP. (C) Clone stained for GFP (green); note that FmiΔIntraEGFP localizes to apical junctions in the absence of endogenous *fmi* (arrowheads). (D) Clone stained for Stbm (blue) and Phalloidin to stain trichomes (red). Trichomes point away from the clone (arrowheads), similar to a *stbm* loss-of-function clone. (E) Clone stained for Stbm (green); white arrowheads point to staining on the clone boundary and yellow arrowheads to asymmetric localization inside the clone. (F) *fmi^E59^* clone, marked by loss of lacZ staining (red) and stained for Stbm (green).

Endogenous Fmi is thought to be localized on both proximal and distal cell boundaries. We confirmed this by expressing CFP-tagged Fmi at physiological levels in clones in pupal wings and observed that levels of staining appear similar at each end of the cell (Figure 2A), consistent with the homophilic-interaction model. Notably, expression of a GFP-tagged form of FmiΔIntra results in its preferential localization to distal cell edges, where Fz and Dsh also localize (Figure 2B, Figure S1D).

Interestingly, junctional localization of FmiΔIntra-EGFP is not dependent on endogenous, full-length Fmi (Figure 2C), suggesting that this molecule is still able to participate in homophilic interactions. Hence, we investigated the ability of FmiΔIntra-EGFP to functionally rescue the polarity phenotype of *fmi* null mutant clones. If FmiΔIntra-EGFP interacts preferentially with the distal Fz:Dsh complex, then Stbm recruitment to junctions inside clones would be compromised. Consequently, FmiΔIntra-EGFP:Fz complexes inside the clone would preferentially interact with Fmi:Stbm outside the clone, leading to a reversal in polarity on proximal clone edges. Importantly, this prediction is upheld, and *fmi* clones rescued with FmiΔIntra-EGFP exhibit weak proximal polarity inversions, such that trichomes point away from the clone (Figure 2D), and polarity proteins are recruited to the clone boundary (white arrowheads in Figure 2E).

Nevertheless, Stbm localizes asymmetrically inside the clone, although not always at the correct site (Figure 2E, yellow arrowheads), whereas in a *fmi* null mutant it lacks any asymmetric localization (Figure 2F) [18]. Thus, FmiΔIntra-EGFP must retain some ability to interact with Stbm. To confirm this, we analyzed the ability of full-length Fmi or FmiΔIntra-EGFP to interact with Fz and Stbm in *Drosophila* S2 cells. In this assay, Fmi and FmiΔIntra-EGFP are recruited to sites of cell contact, as a result of homophilic interactions between their extracellular domains (Figures S2A and S2B) [3, 28]. Cotransfection of Fz or Stbm with either full-length Fmi or FmiΔIntra-EGFP in *Drosophila* S2 cells results in the recruitment of both to sites of cell contact (Figures S2E–S2H).

Interestingly, if S2 cells were transfected with either Fz or Stbm and then mixed, we also observed weak recruitment to sites of cell contact (Figure S2I), arguing that their extracellular domains can interact independently of Fmi. Nevertheless, recruitment was weaker and less frequent than when Fmi was cotransfected, suggesting that Fmi:Fmi interactions are more important than Fz:Stbm interactions in stabilizing complexes between adjacent cells.

### Frizzled and Strabismus:Prickle Are Required in Opposite Cells for Flamingo to Make Stable Homophilic Interactions

**Figure 3:**
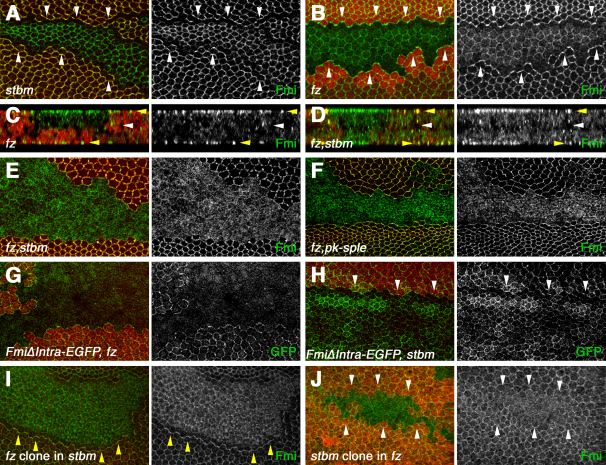
Fmi Localization to Apicolateral Junctions Is Dependent on Fz and Stbm:Pk (A–F) Fmi staining (green) in pupal wing clones, marked by loss of Stbm (A), lacZ (B and C), or Fz-GFP (D–F). White arrowheads in XY sections indicate clone boundaries. (A) *stbm^6^* clone. Fmi levels are reduced at junctions. (B) *fz^21^* clone. Fmi localization at junctions is hazy, and increased at apical membranes. (C and D) XZ sections of *fz^21^* clones (C) or *fz^21^, stbm^6^* double-mutant clones (D). Images show the double cell layer of the pupal wing: Yellow arrowheads indicate the apical surfaces and white arrowheads the apposed basal surfaces. Increased apical staining of Fmi is evident within the clones. (E and F) *fz^21^, stbm^6^* (E) and *fz^21^, pk^pk-sple-13^* (F) double-mutant clones. Fmi is poorly localized to junctions but is distributed throughout the apical membrane. (G and H) *fz^21^* clone (G) or *stbm^6^* clone (H) marked by loss of lacZ (red), in wings expressing FmiΔIntra-EGFP, and stained for GFP (green). (I) *fz^21^* clone, marked by loss of Dsh staining (red), in a *stbm^6^* mutant background. Note that the Dsh antibody gives background staining of nuclei. Fmi (green) is recruited to the clone boundary (yellow arrowheads). (J) *stbm^6^* clone in a *fz^21^* mutant background, marked by loss of lacZ (red), and stained for Fmi (green). White arrowheads indicate clone boundaries.

Previous reports indicate that loss of either Fz or Stbm only has a mild effect on Fmi localization to junctions, although the proximodistal asymmetry is lost. Thus, in *stbm* null mutants, Fmi remains apical, but at slightly reduced levels, and appears to be less tightly associated with junctions (Figure 3A) [18]. In *fz* null mutants, we observe a slightly more severe effect than previously reported for hypomorphic alleles [3, 22], with only hazy localization at junctions (Figure 3B) and an increase in apical staining (Figure 3C). This reduction in tight staining of Fmi at junctions is suggestive of a reduced stability of Fmi homophilic interactions.

In contrast, simultaneous loss of both *fz* and *stbm* activity has a dramatic effect on Fmi localization. Whereas a low level remains associated with junctions (Figure 3E), the majority of Fmi is seen apically (Figures 3D and 3E). Extracellular staining of Fmi in the absence of detergent shows that the majority of this apical Fmi population is located at the plasma membrane, rather than in vesicles close to the cell surface (Figures S3A and S3B). An identical phenotype is observed with *fz, pk* double-mutant clones (Figure 3F). Thus, whereas Fmi is required for apical recruitment of Fz and Stbm, Fmi is also mutually dependent on Fz and Stbm:Pk for apicolateral localization.

Interestingly, whereas full-length Fmi has a partially redundant dependence on Fz and Stbm for junctional localization, FmiΔIntra-EGFP is entirely dependent upon Fz, but not on Stbm (Figures 3G and 3H). This again supports the idea that C-terminally deleted Fmi interacts poorly with Stbm.

Fz appears to be more important in stabilizing Fmi at junctions than Stbm (compare Figure 3A to Figure 3B). This difference was confirmed by experiments in which we induced *fz* clones in a *stbm* mutant background, and vice versa (Figures 3I and 3J). In both cases, Fmi localizes apically in the double-mutant tissue, as expected. However, if *fz* clones are induced in a *stbm* background, then Fmi accumulates on the clone boundaries (Figure 3I, yellow arrowheads), suggesting that Fmi:Fz in one cell is better at stabilizing Fmi in the adjacent cell than Fmi alone. In contrast, if *stbm* clones are induced in a *fz* mutant background, then no accumulation of Fmi on clone boundaries is seen (Figure 3J). Thus, Fmi in one cell is not significantly stabilized by Fmi:Stbm in its neighbor.

These results reveal that Fmi stability at junctions is dependent on proximal and distal complexes to differing degrees, with the most stable conformation being Fmi:Fz in one cell and Fmi:Stbm in the adjacent cell. In the absence of Stbm, a Fz:Fmi complex on one boundary has significant ability to stabilize Fmi at junctions in the adjacent cell, whereas in the absence of Fz, a Stbm:Fmi complex shows negligible stabilization of Fmi in the neighboring cell. Finally, Fmi fails to interact significantly across cell boundaries in the absence of both Fz and Stbm.

### Loss of Frizzled or Strabismus Increases Flamingo Levels in an Endocytic Compartment

Recent experiments have indicated that Fmi and Fz are trafficked on vesicles toward distal cell edges [25]. The number of Fz particles observed is most abundant at the time during which asymmetric localization becomes most pronounced, but particles are seen throughout pupal development, suggesting that the Fmi and Fz are continually being trafficked. Because Fmi is apparently less stable at junctions in the absence of Fz and/or Stbm:Pk, we wondered whether this differential stability correlated with an increase in Fmi endocytic trafficking.

It has previously been observed that inhibiting lysosomal maturation in flies causes a readily observable enlargement of the endosomal compartment [29], in which endocytosed proteins accumulate (e.g., [30]). We predicted that a more rapidly endocytosed protein would spend more time trafficking through the endosomal compartment and thus would accumulate to a greater extent in such an enlarged endosomal compartment, at the expense of the plasma membrane.

**Figure 4:**
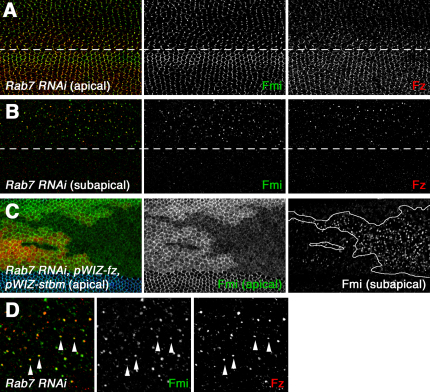
Localization of Fmi to an Endocytic Compartment (A and B) Apical (A) and subapical (B) sections of pupal wings expressing *Rab7* RNAi in the *ptc-GAL4* domain (above white line). Expression of *Rab7* RNAi causes an accumulation of Fmi (green) and Fz (red) in intracellular puncta. No effect on overall protein levels was observed by western blot (not shown). (C) Apical (left and middle) and subapical (right) sections of pupal wings expressing *fz* and *stbm* RNAi and *Rab7* RNAi in the *ptc-GAL4* domain (marked by loss of Stbm in blue). *Rab7* RNAi is expressed in the subset of the *ptc-GAL4* domain where HcRed staining (red) is absent (outlined in white in right panel). Fmi (green) is lost from apical membranes (middle) and accumulates in subapical intracellular puncta (right). The *fz* and *stbm* RNAi transgenes effectively knocked down Fz and Stbm protein levels, as determined by immunolabeling, and reproduced the expected effects on Fmi protein localization (Figure S3A). (D) High-magnification image of subapical sections of pupal wings expressing dominant-negative *Rab7^TN^* in the *ptc-GAL4* domain. Arrowheads indicate colocalization of Fmi (green) and Fz (red) puncta.

To test this, we expressed dsRNA constructs against factors required for lysosomal maturation (reviewed in [31]) in a stripe in the pupal wing. Knockdown of the small GTPase *Rab7* or *hrs* did not affect the asymmetric localization of Fmi and Fz (Figure 4A, top; Figure S4A), but, as expected, they accumulate in enlarged intracellular puncta (Figure 4B, top; Figure S4B). Colocalization of Fmi and Fz was seen (Figure 4D, 80% of Fmi puncta also contained Fz), but there was no localization of Stbm in puncta (Figure S4B).

Next we investigated Fmi trafficking in *fz, stbm* double mutants, where we predict that Fmi would be less stabilized at the plasma membrane and rates of endocytic trafficking would increase. *fz* and *stbm* expression were knocked down with RNAi in a stripe in the center of the wing, and at the same time we expressed *Rab7* dsRNA randomly in clones of cells. In these clones, there is a significant reduction in the population of Fmi at the plasma membrane, and more Fmi accumulates in an increased number of puncta (Figure 4C). Levels of other junctional proteins such as E-cadherin are unaffected in this background (Figure S4C), suggesting that there is no general defect in endocytic trafficking. A subtle decrease in Fmi levels at junctions is also observed when a *Rab7* dominant negative is expressed in *fz* or *stbm* single mutants (Figures S4D and S4E). These data are consistent with our hypothesis that loss of *fz* and *stbm* activity results in an increased rate of endocytic trafficking of Fmi.

### Flamingo and Frizzled Are Targeted to Both Recycling and Degradative Compartments

We then investigated the identity of the endosomal compartment in which Fmi accumulates when dominant-negative *Rab7* or *Rab7* dsRNA is expressed. We observed that Fmi staining colocalizes with or is adjacent to the early endosomal marker Rab5 (reviewed in [31]) (Figure S5A). Furthermore, strong colocalization was seen with Rab4, a small GTPase required for recycling of proteins back to the plasma membrane from the endosome (Figure S5B). No colocalization was seen with Rab11, which mediates recycling by an alternative route (data not shown). Thus, our data suggest that Fmi is present in an endosomal compartment with Rab4 and Rab5 subdomains [32].

In the absence of *Rab7^TN^* expression, small Fmi puncta can also be seen colocalizing with Rab4 (Figure S5C), suggesting that this is its normal trafficking route. However, we saw no effect on Fmi or Fz levels at apicolateral junctions when Rab4 dominant negatives are expressed (Figure S5D). In the absence of Rab4, Rab11 could possibly be used to return Fmi to junctions. Blocking of Rab4 and Rab11 function simultaneously caused a loss of Fmi at junctions; however, it also affected the trafficking of other junctional proteins such as E-cadherin, and so the effects on Fmi may be indirect (Figure S5F). These data are consistent with the hypothesis that Rab4 is normally used to recycle Fmi to junctions, but in its absence an alternative pathway is used.

**Figure 5:**
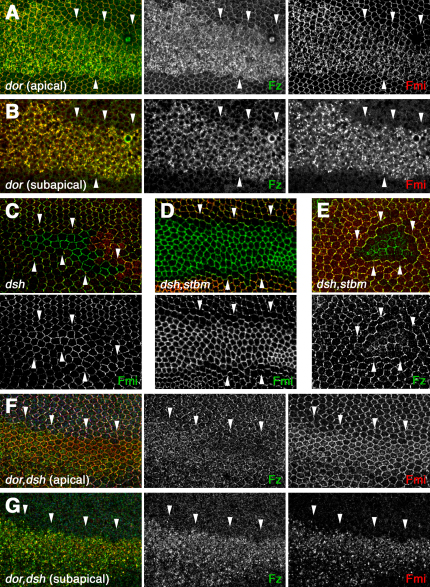
Dsh-Independent Trafficking of Fmi (A and B) Apical (A) and subapical (B) sections of pupal wings expressing unmarked *dor^8^* clones. Fz (green) and Fmi (red) accumulate at apical junctions and intracellularly. (C) *dsh^3^* clone, marked by loss of Dsh staining (red). Fmi (green) is slightly increased at junctions. (D and E) *dsh^3^ stbm^6^* double-mutant clones, marked by loss of Stbm-EYFP (red). Fmi (green in [D]) accumulates at apicolateral junctions, whereas Fz (green in [E]) does not. (F and G) Apical (F) and subapical (G) sections of pupal wings expressing *dor^8^ dsh^3^* double-mutant clones, marked by loss of Dsh (blue). Fz (green) and Fmi (red) still accumulate intracellularly. White arrowheads indicate clone boundaries.

We also investigated whether the degradative pathway is important in regulating the subcellular distribution of the polarity proteins. Although expression of *Rab7* dsRNA causes some intracellular accumulation of Fmi and Fz, the block in lysosomal maturation is unlikely to be complete. Therefore, we made null mutant clones of *deep orange* (*dor* or *Vps18*), which is essential for lysosomal maturation [33]. In these clones, very large amounts of Fmi and Fz (but again not Stbm) accumulate intracellularly (Figures 5A and 5B). Furthermore, we sometimes see an increased level of Fmi at junctions, consistent with greater recycling of intracellular Fmi to the junctional region. Therefore, we surmise that a proportion of Fmi and Fz entering the endocytic pathway is normally targeted for lysosomal degradation.

### Flamingo Is Trafficked via Dishevelled-Dependent and Dishevelled-Independent Mechanisms

As we have already noted, blocking of intracellular trafficking results in colocalization of Fz and Fmi in intracellular puncta. Similarly, Shimada et al. [25] presented evidence that Fz and Fmi were coendocytosed and transported toward the distal cell edge. However, the signals promoting Fmi internalization presumably vary, depending on whether Fmi is complexed with other polarity proteins either within a cell or across junctions. In particular, we hypothesize that there may be specific mechanisms for removing Fmi that is not in stable asymmetric complexes from junctions, explaining the negligible levels of junctional Fmi in *stbm, fz* double mutants.

Previous studies have shown that Fmi levels at apical junctions are slightly increased in *dsh* mutants (Figure 5C) [21], without any corresponding change in Fz levels [22]. Thus, Dsh may normally promote the removal of Fmi that is not in asymmetric complexes from junctions, consistent with its reported action as an endocytic adaptor in other contexts [26, 27].

In *dsh, stbm* double-mutant clones, we saw an even more striking junctional accumulation of Fmi (Figure 5D), but again no increase in Fz (Figure 5E). This suggests that Stbm also promotes the removal of noncomplexed Fmi from junctions, in addition to its role in stabilizing Fmi by participating in the formation of asymmetric complexes with Fz in neighboring cells (see Discussion).

Because Fmi accumulates at junctions in a *dsh* or *dsh, stbm* double-mutant background, but Fz does not, we conjectured that Dsh and Stbm promote trafficking of Fmi when it is not in a complex with Fz. To test this, we investigated the trafficking of Fmi and Fz in *dor, dsh* double-mutant clones. If Dsh promoted the cotrafficking of Fmi and Fz, then the coaccumulation of Fmi and Fz in *dor* mutant clones would be blocked in the absence of Dsh. Alternatively, if Dsh only promoted Fmi endocytosis when Fmi was not associated with Fz, then this coaccumulation would not be predicted to alter. Notably, Fmi and Fz accumulate intracellularly to a similar extent in *dor, dsh* double-mutant clones and in *dor* single-mutant clones (Figures 5F and 5G). A similar result was seen with *dor, dsh, stbm* triple-mutant clones (data not shown). This confirms that Fmi that is associated with Fz is internalized and targeted to the degradative pathway in a Dsh- and Stbm-independent fashion.

## Discussion

Our data suggest that Fz:Dsh and Stbm:Pk complexes differ in their ability to associate with Fmi. Whereas endogenous levels of Fmi result in the formation of asymmetric complexes with Fz:Dsh on one side of the boundary and Stbm:Pk on the other, overexpressing Fmi favors Fz:Dsh recruitment. Furthermore, a C-terminally deleted form of Fmi preferentially localizes distally with Fz, and overexpression of this form has an even greater preference for Fz:Dsh recruitment. Thus, the C terminus of Fmi is important in promoting the interaction with Stbm:Pk. The Fmi truncation data could be explained simply by the possibility that the C terminus of Fmi contains a direct binding site for Stbm; however, this fails to explain why overexpressed full-length Fmi prefers to recruit Fz:Dsh. We therefore propose that the association of Fmi with Stbm:Pk requires a limiting factor that is saturated by Fmi overexpression. The most plausible hypothesis is a requirement for a cofactor for Stbm:Pk binding, but other possibilities include saturation of the machinery for a posttranslational modification or a conformational change in Fmi.

Our data also suggest that Fmi itself needs to associate with both proximal and distal components in order to be stably localized to apicolateral junctions. Although it can form homophilic dimers between adjacent cell membranes in tissue culture, in pupal wings Fmi does not localize strongly to apical junctions and presumably fails to form stable homodimers in *trans*. We find that Fz on one side of the junction and Stbm:Pk on the opposite side stabilize Fmi at junctions, most likely by promoting homophilic interactions or preventing internalization. However, Fmi appears to be capable of forming complexes with either distal or proximal components alone, but these complexes (particularly the proximal complex) are apparently less stable at junctions. Taken together with our overexpression experiments, this would suggest that the most stable configuration is Fz:Fmi on one side of the boundary and Fmi^∗^:Stbm:Pk on the other (where Fmi^∗^ denotes the modified form able to preferentially associate with Stbm:Pk).

In order for an asymmetric complex to be stabilized across junctions, the extracellular domains must somehow “look” different. One possibility is that the Fz and Stbm extracellular loops interact—a view supported by our S2 cell data. Alternatively, the Fmi extracellular domain, when associated with either Fz or Stbm:Pk, could undergo a conformational change that promotes homophilic Fmi interactions.

An intriguing question is why clones of cells that overexpress Fmi behave like *fz* loss-of-function clones [3]. We suggest that within the clones, excess Fmi associates with the entire available pools of both Fz and Stbm. However, there is still a pool of uncomplexed Fmi that can associate with Fmi:Fz in adjacent wild-type cells, forming the relatively stable Fmi-Fmi:Fz configuration, thus causing polarity to be reversed on distal clone boundaries. In support of this model, an identical nonautonomous effect is seen when FmiΔIntra is overexpressed (Figure S6), which itself interacts only poorly with Stbm but presumably can interact with Fmi:Fz in adjacent cells outside the clone.

Interestingly, Fmi accumulates in excess at junctions in a *dsh, stbm* double mutant, whereas Fz does not. Thus, although Fz acts to stabilize Fmi at junctions, Fmi does not always need to associate with Fz in a stoichiometric fashion in order to be stabilized. Perhaps as long as there is some Fz associated with Fmi, this may permit local stabilization of other Fmi molecules in *cis*. Alternatively, this excess accumulation of Fmi might simply represent “unstable” Fmi homodimers that are no longer being removed from junctions by the actions of Dsh and Stbm.

The composition of the complex with which Fmi is associated appears to be critical for determining the frequency and manner by which Fmi is turned over from the plasma membrane. Most compellingly, Fmi accumulates more strongly in an enlarged endosomal compartment in *Rab7^TN^* mutant tissue when *stbm* and *fz* are absent than when they are present. Thus, we suggest that more Fmi is resident in the endocytic pathway when it is unable to form stable asymmetric complexes. Fmi:Fz puncta have previously been observed that are selectively trafficked to distal cell edges [25]. In our experiments, these puncta colocalize with YFP-Rab4, suggesting that Fmi and Fz are recycled back to the plasma membrane by a Rab4-dependent mechanism. Furthermore, the increased intracellular and junctional levels of Fz and Fmi in *dor* mutant clones suggests that in addition to being recycled to the plasma membrane, a significant fraction of internalized Fmi and Fz is also sent for degradation. It is formally possible that the intracellular accumulation of Fmi and Fz seen when lysosomal trafficking is blocked by loss of *Rab7* or in *dor* clones is due to their being sent for degradation immediately after synthesis (e.g., if damaged or misfolded); however this is unlikely because newly synthesized Fmi-ECFP appears first at junctions before been seen in puncta (Figures S4F and S4G).

We have never observed Stbm in large intracellular puncta, but it seems likely that it is also internalized and recycled, possibly together with Fmi, although it must do so by alternative pathways involving smaller or more rapidly recycling particles that are not visible by confocal microscopy. Indeed, our data suggest a potential role for Dsh and Stbm in regulating junctional levels of Fmi. A *stbm* mutant alone results in a loss of Fmi from junctions, consistent with a need for Stbm in stabilizing Fmi in asymmetric complexes. In contrast, loss of Dsh and Stbm together increases Fmi levels at junctions, suggesting a role for Stbm in internalization. We would suggest that the outcome of any interaction of Stbm with Fmi is dependent upon whether Fmi is able to form stable homodimers with Fz on the opposite cell membrane. In a wild-type situation, one could envisage that Fmi forms stable homodimers in a Fz:Fmi-Fmi^∗^:Stbm configuration, and that both Dsh and Stbm promote internalization of any Fmi that is not in this configuration, the majority of which is subsequently recycled back to the plasma membrane. In *dsh* mutants, there is reduced internalization, but the effect on Fmi levels is subtle; Fz and Stbm are still present to promote Fmi homodimer formation, and Stbm still promotes internalization of any unstable Fmi. In contrast, in *stbm* mutants, the number of less stable Fmi complexes (associating only with Fz) is greatly increased, favoring internalization by Dsh. Finally in *dsh, stbm* double mutants, Fmi is again less stable (associating only with Fz), but there is no Dsh- or Stbm-mediated internalization, leading to an overall increase of Fmi at junctions.

How do Dsh and Stbm regulate Fmi levels at junctions? Stbm contains potential interaction motifs for the endocytic adaptor AP2 (Figure S7) [34], but their role has not been functionally tested. In addition, in vertebrate Wnt signaling, there is evidence that Dsh interacts with the endocytic adaptor protein β-arrestin and the μ2 subunit of AP2 [26, 27] to mediate Wnt/Fz endocytosis and downregulation of Wnt signaling. Interestingly, in planar polarity we do not have any evidence that Dsh directly mediates internalization of Fz, but our data rather point to Dsh promoting Fmi internalization when it is not associated with Fz. Instead, the trafficking of Fmi together with Fz into the lysosomal pathway is Dsh independent.

In summary, we propose that a number of mechanisms exist by which Fmi contributes to the generation of asymmetry at the molecular level. First, our characterization of the previously inferred asymmetry in Fmi activity indicates that Fmi normally prefers to bind to Fz and requires a limiting factor for association with Stbm:Pk. Second, Fmi stability at junctions is dependent on both Fz and Stbm:Pk, with the most stable form being Fz:Fmi bound to Fmi^∗^:Stbm. Finally, we propose that entry of Fmi into the endocytic trafficking pathway is decreased if it is in a stable complex, and this is regulated either by Dsh and Stbm or independently of Dsh and Stbm, depending on whether it is associated with Fz.

An outstanding question is how these mechanisms translate into cellular asymmetry, such that in any particular cell, heterophilic polarity complexes preferentially form with Fz:Dsh at the distal junctions, rather than having heterophilic complexes in both orientations. We think that the acquisition of cellular asymmetry is likely to be driven by directional trafficking of Fmi:Fz [25], although other models, such as a mechanism for preferential stabilization of Fmi:Fz interactions at the distal cell edge, are also possible. In addition, it seems likely that an amplification mechanism would be required [4, 5, 17, 22], although the molecular mechanisms remain to be elucidated.

While this manuscript was in preparation, another manuscript was published, in which Fmi was proposed to mediate an asymmetric and instructive signal between proximal and distal complexes to generate asymmetry [35], and thus does not act merely as a scaffold for Fz:Stbm interactions across membranes. We would argue that our data do not provide evidence for a specific signaling function of Fmi. Instead, we favor the hypothesis that the composition of the proximal and distal complexes is distinct, and that heterophilic complexes are inherently more stable than homophilic complexes. Together, removal of unstable nonasymmetric complexes through increased endocytic turnover, in concert with directional trafficking and an unknown amplification mechanism, may be sufficient to generate asymmetry without the need to invoke a specific signaling function for any components of the complexes.

## Experimental Procedures

Alleles and transgenes are described in FlyBase, except for *pWIZ-fz* and *pWIZ-stbm* [36]; *P{w+, Casper-tub-YFP-Rab4}* and *P{w+, Casper-tub-CFP-Rab5}* [37]; and *P{w+, UAS-FmiΔIntra}*, *P{w+, ActP-FRT-PolyA-FRT-Fmi-ECFP}* and *P{w+, ActP-FRT-PolyA-FRT-FmiΔIntra-EGFP}* (this study). *UAS-Fmi* expresses the *fmi* isoform that lacks the PBM [3]. The *hrs* and *Stam* RNAi flies were obtained from the VDRC. Mitotic clones were generated with the FLP/FRT system [38] and *Ubx-FLP* [39]. Overexpression was carried out with the GAL4/UAS system [40]. Expression from *pUHR-Rab7^TN^*, *pFRIPE-Rab7*, *pUHR-Rab4^SN^*, and *pFRIPE-Rab11* was induced by 2 hr heatshocks in 1^st^- and 2^nd^- instar larvae, to flip out the *FRT-HcRed-FRT* cassette [37], and fly lines with a permanent excision of the *FRT-HcRed-FRT* cassette were also made for *Rab4^SN^*, *Rab7^TN^*, and the *Rab7* dsRNA construct. Double-mutant clones were generated as previously described [16].

Pupal wings were aged at 25°C and dissected at 28 hr after prepupa formation (APF) or 32 hr APF for trichomes, and wings were processed for immunofluorescence and imaged as previously described [22]. Primary antibodies used were mouse monoclonal anti-ßgal (Promega), rabbit anti-ßgal (Cappel), rabbit anti-GFP (Abcam), mouse monoclonal anti-Arm (DSHB), rat monoclonal anti-E-cadherin (DSHB), mouse monoclonal anti-Fmi#74 (DSHB) [3], rabbit anti-Fz [36], rat anti-Dsh [41], and rabbit anti-Pk [5]. The Stbm rat antibody was directed against a His-tagged fusion protein corresponding to amino acids 406–584. Actin was visualized with Alexa568-conjugated phalloidin (Molecular Probes). Extracellular staining was carried out in the absence of detergent.

FmiΔIntra and FmiΔIntra-EGFP contain a deletion of the entire intracellular domain of Fmi, except for the first 30 amino acids after the last transmembrane domain, which are retained. Fmi-ECFP is a fusion of the coding sequence of CFP in frame to the last amino acid of Fmi, and FmiΔIntra-EGFP is a fusion of GFP to the C terminus of FmiΔIntra. Constructs were cloned into *P{w+, UAST}* [40] or *P{w+, ActP-FRT-PolyA-FRT-PolyA}* [22], and germline transformations were carried out. Fmi-FLAG [16], Fmi-ΔIntra-EGFP, Stbm-EYFP [42], and Fz were also cloned into the tissue-culture transformation vector pMK33 for transfection into S2 cells with Effectene (QIAGEN).

## Supplemental Data

Document S1. Seven Figures
