# Supplementary material for: Differential Stability of Flamingo Protein Complexes Underlies the Establishment of Planar Polarity
Source: Curr Biol. 2008 Oct 28;18(20-3):1555–64. doi: 10.1016/j.cub.2008.08.063 (PMC2593845; doi:10.1016/j.cub.2008.08.063)
Supplement: Document S1. Seven Figures [file mmc1.pdf]

Current Biology, Volume *18*

Supplemental Data

Differential Stability of Flamingo Protein Complexes Underlies the Establishment of Planar Polarity

Helen Strutt and David Strutt

Supplemental Figures

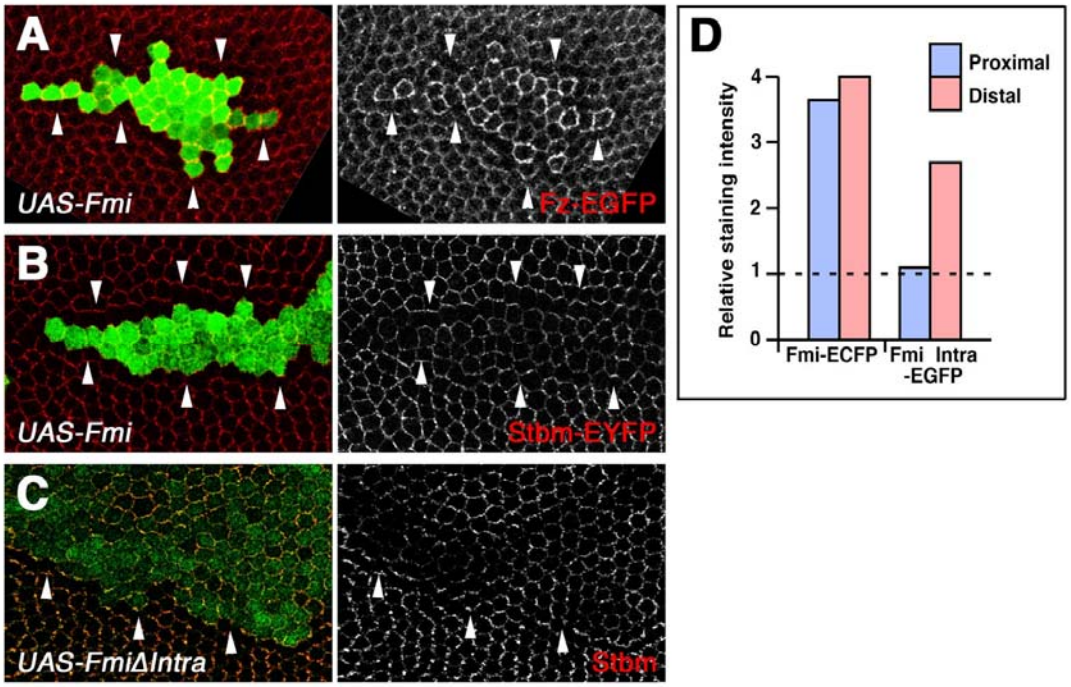

Figure S1. Overexpression of Fmi.

(A-B) Pupal wing clones overexpressing Fmi, marked by lacZ staining in green. Wings are expressing Fz-EGFP under control of the Armadillo promoter [1] (A) or Stbm-EYFP under control of the Actin promoter [2] (B), and stained for GFP in red. (C) Pupal wing clones overexpressing FmiΔIntra, marked by Fmi staining in green, and stained for Stbm in red. Arrowheads indicate clone boundaries. (D) Quantitation of Fmi-ECFP or FmiΔIntra-EGFP staining at proximal (blue) and distal (red) membranes. Mean immunofluorescence on proximal and distal cell boundaries of at least 40 isolated cells was quantitated in Image SXM, and normalised to the mean immunofluorescence in the cytoplasm, indicated by the dashed line.

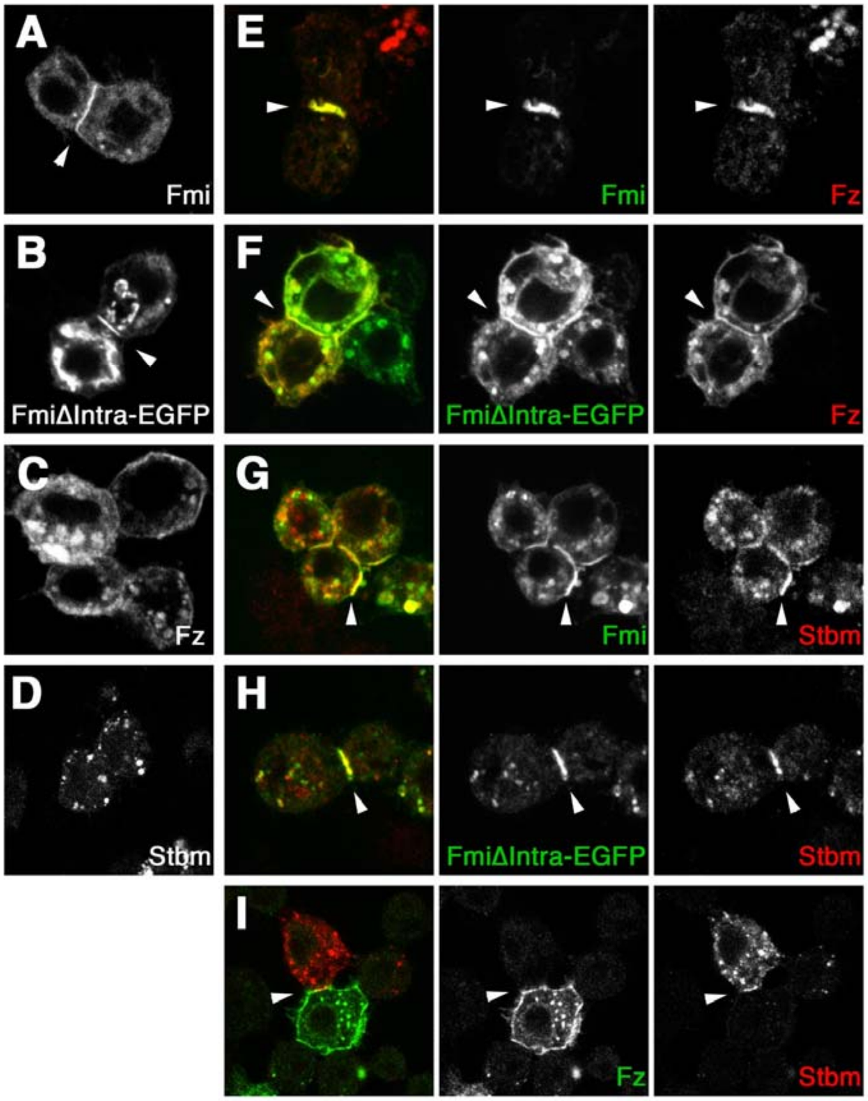

Figure S2. Recruitment of Fz and Stbm to cell boundaries by Fmi and FmiΔIntra-EGFP in S2 cells.

Transient transfections of S2 cells. (A, B) Cells transfected with Fmi-FLAG (A) or FmiΔIntra-EGFP (B), and stained for Fmi. Fmi is recruited to boundaries between adjacent cells (arrowheads). (C, D) Cells transfected with Fz (C) or Stbm-EYFP (D), and stained with Fz or Stbm antibodies. There is no preferential recruitment to cell-cell boundaries. (E-H) Cells co-transfected with Fmi (E, G) or FmiΔIntraEGFP (F, H), together with Fz (E, F), Stbm-EYFP (G, H), and stained with the appropriate antibodies. Fz and Stbm are recruited by Fmi to cell-cell boundaries (arrowheads). (I) Cells transfected with either Fz or Stbm and then mixed. Some cells show recruitment to cell-cell boundaries (arrowhead).

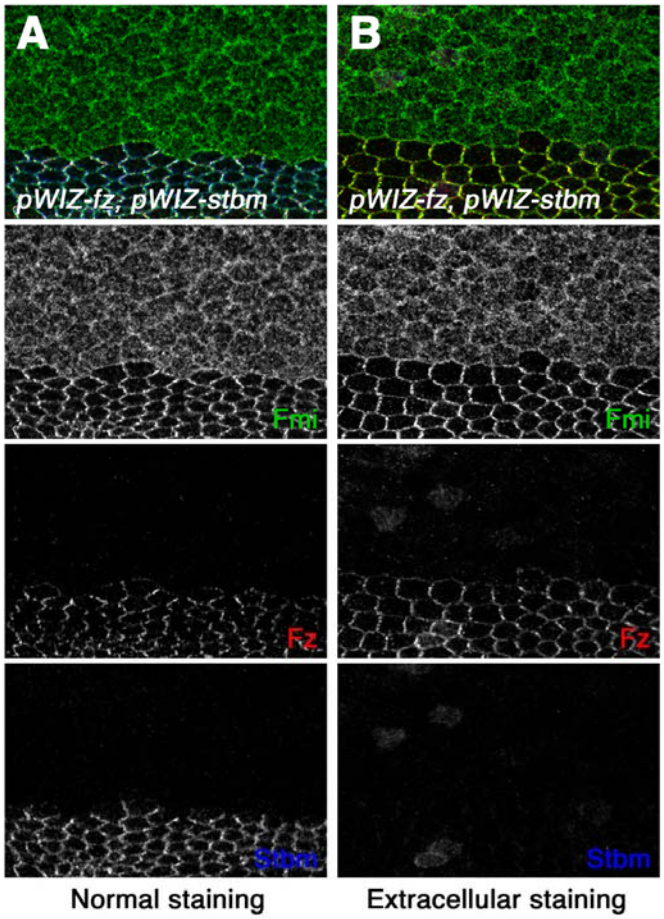

Figure S3. Normal and extracellular staining of Fmi in pupal wings expressing *fz* and *stbm* RNAi.

Pupal wings expressing *fz* and *stbm* RNAi in the *ptc-GAL4* domain (top part of panels), stained by a normal staining procedure (A) or in the absence of detergent for extracellular staining (B). The Fz antibody recognises an extracellular epitope and can be visualised with both staining protocols (red), whilst the Stbm antibody recognises an intracellular epitope and can only be seen with the normal staining procedure (blue). The Fmi extracellular epitope can be seen apically (green) using both staining procedures, suggesting it is at apical membranes and not in apical puncta.

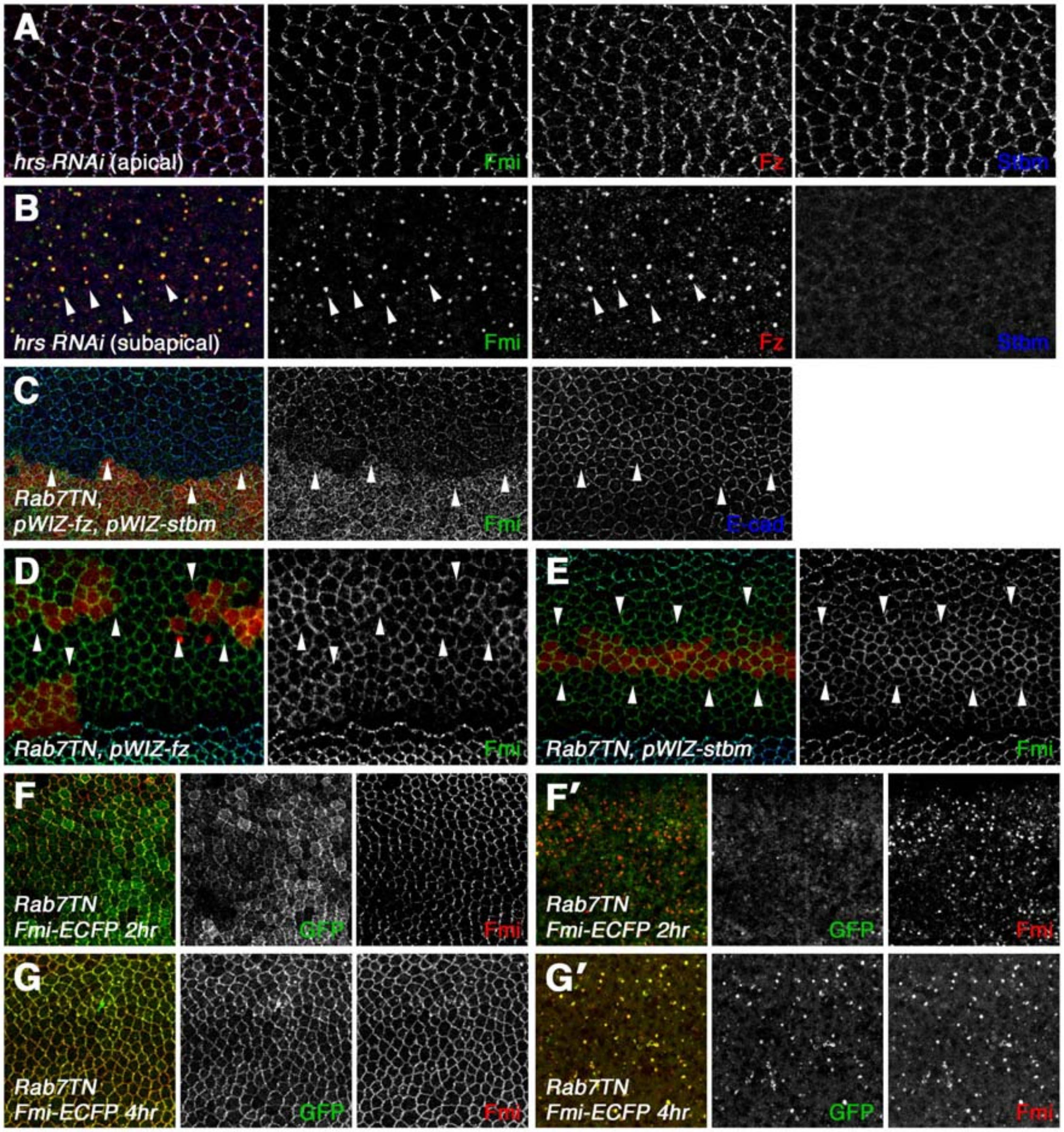

Figure S4. Expression of *hrs* RNAi and Rab7^{TN}.

(A, B) Apical (A) and sub-apical (B) sections of pupal wings expressing dsRNA against *hrs* and stained for Fmi (green), Fz (red) and Stbm (blue). Asymmetric localisation at apicolateral junctions is unaffected (A). Fmi and Fz accumulate in intracellular puncta (arrowheads), but Stbm does not (B). (C-E) Apical sections of wings expressing dominant negative *Rab7^{TN}*, and dsRNA against *fz* and *stbm* (C), *fz* dsRNA (D) or *stbm* dsRNA (E), in the *ptc-GAL4* domain. *Rab7^{TN}* is expressed in the subset of the ptc-GAL4 domain where HcRed staining (red) is absent (boundary marked by arrowheads). (C) In the *Rab7^{TN}, pWIZ-fz, pWIZ-stbm* knockdown, Fmi (green) is reduced at apical membranes, whilst E-cadherin (blue) is unaffected. (D, E) A subtle reduction in levels of junctional Fmi is seen when *Rab7^{TN}* is expressed with either *pWIZ-fz* or *pWIZ-stbm*. Note that arrowheads point to the clone boundary, where *Rab7^{TN}* is not expressed. (F, G) Pupal wings expressing *Rab7^{TN}*, dissected 2hr (F) or 4hr (G) after induction of expression of Fmi-ECFP by activation of hs-FLP, and stained for GFP (green) and Fmi (red). Fmi-ECFP has reached apicolateral junctions 2hr after induction of expression (F), but it is not in puncta (F'). 4hr after expression is induced, Fmi-ECFP is at junctions (G) and in puncta (G').

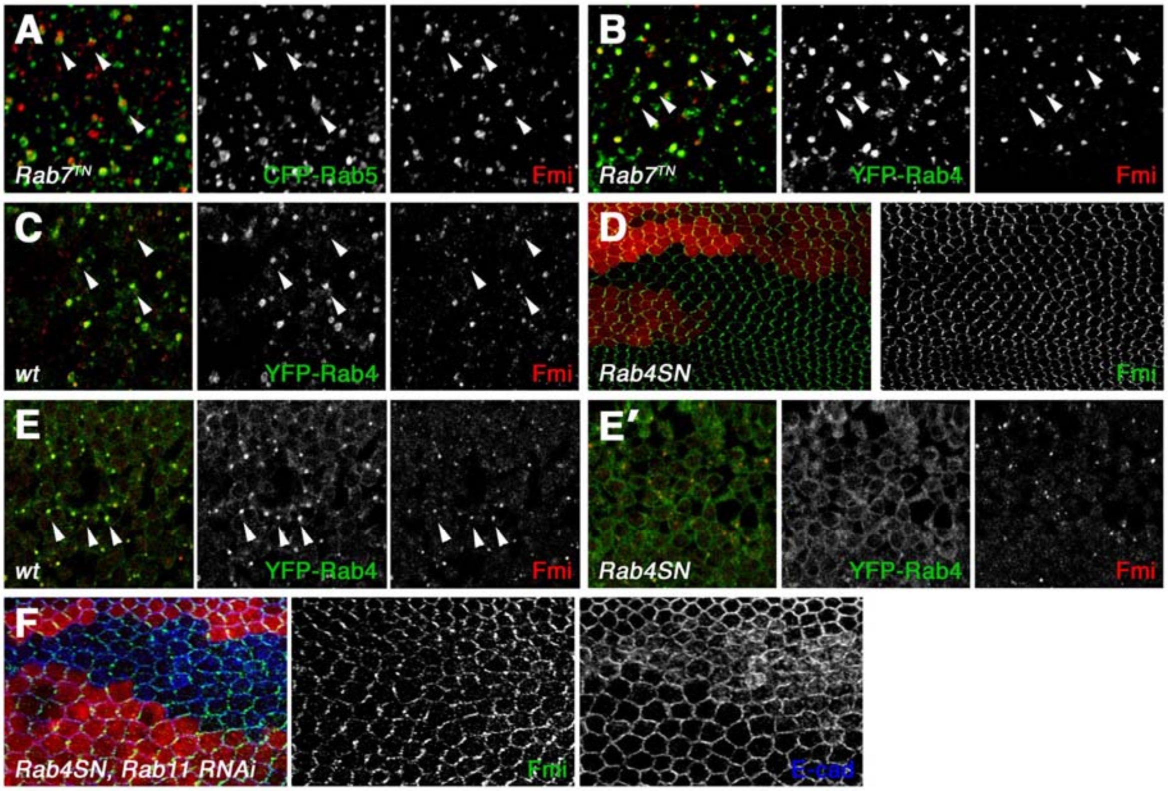

Figure S5. Fmi localises to a Rab4/Rab5 endosomal compartment.

(A-C) Wings expressing $Rab7^{TN}$ in the *ptc-GAL4* domain (A, B) and *tub-CFP-Rab5* (A) or *tub-YFP-Rab4* (B, C) [3]. (A) CFP-Rab5 (GFP staining, green) and Fmi (red) puncta have partial co-localisation (arrowheads). (B, C) Good colocalisation (arrowheads) of YFP-Rab4 (GFP staining, green) and Fmi (red). (D) Wings expressing $Rab4^{SN}$ in the *ptc-GAL4* domain. Expression of $Rab4^{SN}$ (loss of HcRed) does not affect Fmi junctional localisation. (E) Control for $Rab4^{SN}$ activity. Pupal wings co-expressing $Rab4^{SN}$ in the *ptc-GAL4* domain and *tub-YFP-Rab4*, stained for GFP (green) and Fmi (red). YFP-Rab is seen in puncta that co-localise with Fmi outside of the *ptc-GAL4* domain (arrowheads in E), whilst YFP-Rab4-expressing puncta are dispersed when $Rab4^{SN}$ is expressed (E'). (F) Wings expressing $Rab4^{SN}$ and *Rab11* RNAi in the *ptc GAL4* domain. Asymmetric localisation of Fmi (green) is disrupted when both $Rab4^{SN}$ and *Rab11* RNAi are expressed (loss of HcRed), but the distribution of E-cadherin (blue) is also aberrant. No effect is seen if *Rab11* RNAi alone is expressed (not shown), although expression of the dominant negative $Rab11^{SN}$ causes rapid cell toxicity (not shown) [3].

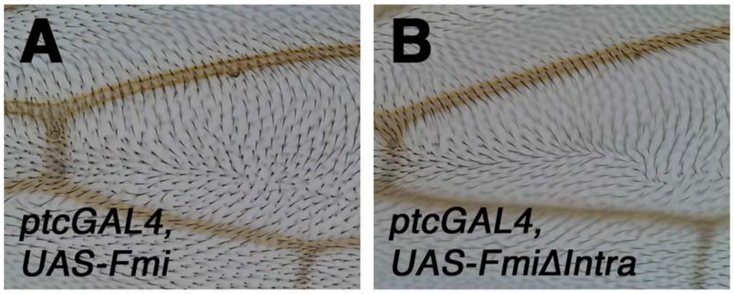

Figure S6. FmiΔIntra overexpression phenotypes

Wings overexpressing full-length Fmi (A) or FmiΔIntra (B), under control of the *ptc-GAL4* driver. In both cases, hairs swirl towards the anterior-posterior boundary of the wing.

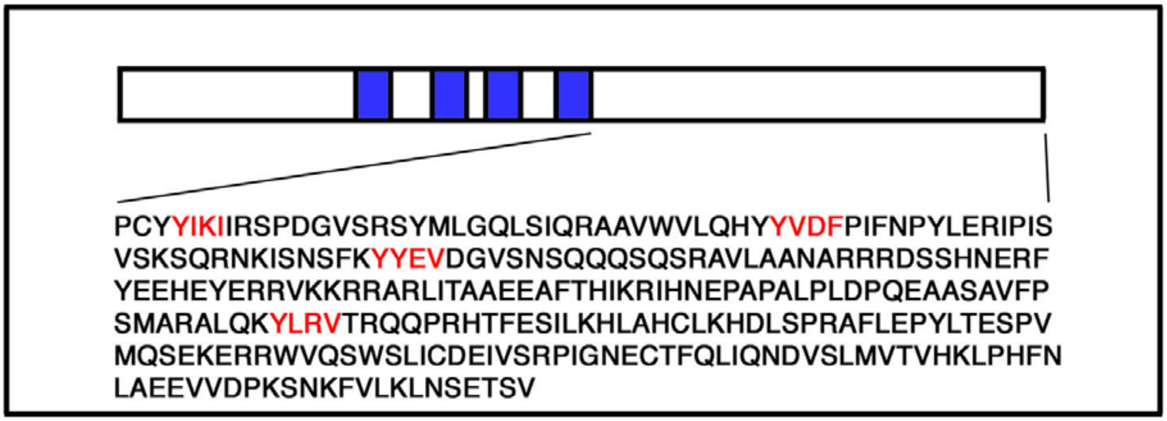

Figure S7. AP2 interaction motifs in Stbm

The entire Stbm ORF is indicated by the bar, and the 4 transmembrane domains are in blue. The sequence of the intracellular C-terminal domain is indicated below. In red are the AP2 interaction motifs Yxxø [4].

Supplemental References

1. Strutt, D.I. (2001). Asymmetric localisation of Frizzled and the establishment of cell polarity in the *Drosophila* wing. Mol. Cell *7*, 367-375.
2. Strutt, D., Johnson, R., Cooper, K., and Bray, S. (2002). Asymmetric localisation of Frizzled and the determination of Notch-dependent cell fate in the *Drosophila* eye. Curr. Biol. *12*, 813-824.
3. Marois, E., Mahmoud, A., and Eaton, S. (2006). The endocytic pathway and formation of the Wingless morphogen gradient. Development *133*, 307-317.
4. Owen, D.J., Collins, B.M., and Evans, P.R. (2004). Adaptors for clathrin coats: Structure and function. Annu. Rev. Cell Dev. Biol. *20*, 153-191.
